# Real-Time MRI Monitoring of Liquid Embolic Agent (Onyx) Injection in a Swine Arteriovenous Malformation Model

**DOI:** 10.3390/brainsci13060915

**Published:** 2023-06-06

**Authors:** Michał Zawadzki, Miłosz Pinkiewicz, Mateusz Pinkiewicz, Jerzy Walecki, Piotr Walczak, Dominika Gołubczyk, Maria Sady, Zdzisław Gajewski

**Affiliations:** 1 Department of Radiology, Centre of Postgraduate Medical Education, 01-813 Warsaw, Poland; 2 Division of Interventional Neuroradiology, Department of Radiology, The National Institute of Medicine of the Ministry of Interior and Administration, 02-507 Warsaw, Poland; 3 Faculty of Medicine, Wroclaw Medical University, 50-368 Wrocław, Poland; 4 Department of Diagnostic Imaging, Mazowiecki Regional Hospital in Siedlce, 08-110 Siedlce, Poland; 5 Program in Image Guided Neurointerventions, Department of Diagnostic Radiology and Nuclear Medicine, University of Maryland, Baltimore, MD 21201, USA; 6 Center for Translational Medicine, Warsaw University of Life Sciences, 02-787 Warsaw, Poland

**Keywords:** real time MRI monitoring, AVM, embolisation

## Abstract

The paradigm is gradually shifting, with radiosurgery and endovascular embolization being increasingly chosen over surgical resection in the selected cases of brain arteriovenous malformations. Routinely used X-ray monitoring of liquid embolic infusion has very good spatial and temporal resolution but is not without significant drawbacks regarding poor visualization of the complex AVM angioarchitecture, especially after many embolizations in the past and therefore limiting the technical ability of the embocure—total occlusion of the feeding arteries, nidus, and draining veins. The purpose of this study was to evaluate the use of real-time MRI guidance in endovascular embolization with Onyx (instead of X-ray) in a single swine rete mirabile (RM) AVM model in order to provide the scaffolding for the real-time MRI guidance method. Onyx propagation was observed in real-time dynamic GE-EPI scan with initial ipsilateral RM filling followed by main cerebral arterial branch distribution. The relatively bright signal within RM and the brain prior to Onyx injection provided a good background for the dark, low signal of the embolic agent spreading in rete mirabile and brain arteries. X-ray picture confirmed Onyx cast distribution at the end of the procedure. In this initial experience, real-time MRI seems to be a promising method that may significantly improve liquid embolic agent infusion monitoring in the future, although requiring further development before clinical use.

## 1. Introduction

Constituting a rare group of complex vascular lesions, brain arteriovenous malformations (bAVM), can have devastating implications on a patient’s quality of life for the risk of intracerebral hemorrhages, seizures, and neurological decline [1,2,3,4]. Therefore, endovascular embolization constitutes an indispensable part of multidisciplinary bAVM management and can be employed for various purposes. Although it has been primarily used preoperatively to support radiosurgery and microsurgery, the substantial advances in catheter technology have significantly improved the safety and efficacy of endovascular embolization of bAVMs [1,2,3,4,5]. Even though the dominant number of neurosurgical centers continues to rely on microsurgery and radiosurgery in the majority of cases, neurointerventional centers effectively employ endovascular embolization as a single curative treatment of bAVMs smaller than 3 cm, located superficially in non-eloquent areas, and with fewer, larger, and less tortuous feeding arteries [1,2,3,4,5]. Moreover, although the results of large clinical trials have yet to be published, transvenous embolization of arteriovenous malformations is emerging as an effective technique with a high rate of total AVM occlusions [6,7]. However, as much as AVM embolization can safely and effectively cure low-grade AVMs, as well as alleviate manifestations in untreatable lesions, it is not a perfect method and requires further improvement [8]. A recent meta-analysis involving 598 AVMs treated with intent-to-cure embolization reported an overall clinical complication rate of 24.1% [9,10]. More importantly, complete obliteration immediately postembolization, equivalent to full treatment, is reported in only 58.4% of bAVM patients [9,10]. These results can be attributed to specific angioarchitectural characteristics of bAVMs (e.g., eloquent cortical location or exclusive deep venous drainage) or technical-related challenges, such as those related to X-ray-based navigation and migration of the embolic material [7,8,9,11]. Being available for almost 100 years, the X-ray constitutes a golden standard for neurointerventions for its ability to delineate a vessel’s lumen and depict devices [9,12]. The X-ray monitoring of endovascular interventions offers great real-time spatial and temporal resolution for the navigation of the devices in the vessels, visualization of contrast media, embolic agents, and metal implants [9,12]. Nevertheless, it also has some considerable limitations [12,13,14,15]. It is projection-based technology with limited soft tissue visualization [12]. DSA images visualize brain tissue as a flow of the contrast media in the parenchymal phase. Cone beam CT produces volumetric, low signal-to-noise ratio static (not real-time) brain images. Any repetition of these images is with great expense of radiation dose [12,13,14,15]. Even with the latest biplane technology, we cannot see through a large embolic agent (e.g., Onyx) cast due to the high level of opacification of the tantalum powder. On the other hand, MRI’s ability to precisely visualize AVMs and depict areas of continuous flow in previously embolised malformations has been widely used in radiotherapy and gamma-knife planning for many years [16]. The latest advances in MRI technology go far beyond the static diagnostic cross-sectional images we are used to in diagnostic neuroradiology [17,18]. Real-time MRI can visualize the movement, structure, and physiology of the tissue at the time it is happening. Images can now be acquired with a speed of up to 50 frames per second—much faster than in the case of fluent fluoroscopy [17,18]. This technology is mostly driven by the development of the imaging of the heart, whose rapid movement had been an obstacle for precise MRI imaging for decades and now can also be used in interventional neuroradiology [17,18]. Navigation of the catheters to the heart is possible using real-time MRI thanks to the active and passive markers mounted on the metal braiding-free catheters [19,20]. Cardiologists are now able to perform the whole procedure of heart ablation under real-time MRI guidance without using X-rays for catheter navigation and procedure monitoring [19,20]. In neuroradiology, microcatheter placement in the brain vessels still requires using X-rays in our model, but there is a lot of ongoing effort to overcome this obstacle.

Aiming to demonstrate the feasibility of MRI guidance, we present this preliminary report on using real-time MRI for visualizing embolic agent—Onyx propagation in the swine rete mirabile and cerebral arteries that resemble human brain AVM angioarchitecture. Because of its similarity to the vascular network of a plexiform human AVM, numerous researchers chose the swine RM as an ideal model for the development of endovascular techniques [21,22,23,24,25,26,27,28,29]. Found at the cranial base, RM is a dense vascular network with plexiform anastomosis between both hemispheres [21,22,23,24,25,26,27,28,29]. Each rete is supplied by the ascending pharyngeal artery, which is a branch of the common carotid artery and the internal carotid artery [21,22,23,24,25,26,27,28,29].

## 2. Materials and Methods

### 2.1. Experimental Animal

One juvenile (3-month-old) female domestic pig (strain PIC, weight 49 kg) was used in this experiment. The animal was acclimated for two weeks at the housing facility before initiating the procedure. The animal had access to water and food ad libitum. Initially, we planned to include five to ten animals in this study. However, in light of the emergence of AVM in vitro models potentially suitable for the evaluation of real-time MRI monitoring of Onyx injection, such as those recently introduced by Kaneko et al., we decided after the first procedure that relying on in vivo models might be unnecessary at this stage [16].

### 2.2. Procedure Description

Surgery was performed in a dedicated large animal surgical suite. The animal was pre-anesthetized with atropine (0.05 mg/kg i.m., Polfa, Poland), xylazine (3 mg/kg i.m., Vet-Agro, Poland), and ketamine (6 mg/kg i.m., Biowet—Puławy, Poland) and anesthetized by propofol (5 mg/kg/h i.v., B.Braun Melsungen AG, Germany) and sevoflurane (1–3%, Abbvie, Poland). The animal received butorphanol every 4 h (0.2 mg/kg i.m., Zoetis, Poland). The femoral artery was identified and punctured using ultrasonography. Using the femoral 6F access, an MRI-compatible, non-braided diagnostic catheter (5F vert—Balton) was navigated under X-ray to the right common carotid artery. The catheter tip was placed at the origin of the right ascending pharyngeal artery. Apollo microcatheter, 3 cm tip, was navigated distally to this artery and placed just proximal to rete mirabile (RM). Continuous flushing with heparinized (5000 U/L) saline was maintained to avoid occlusion of the microcatheter and guiding catheter. All vital parameters (saturation, heart rate, pressure, respiratory rate) were monitored during the entire procedure.

**Figure 1:**
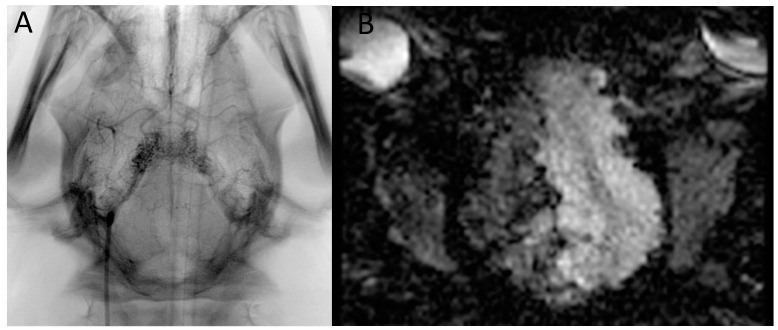
Test infusions through Apollo microcatheter under (**A**) X-ray and (**B**) real-time dynamic EPI-GRE MRI monitoring—dynamic images available in the Supplementary Material Video S1.

DSA run confirmed the filling of the RM and right-sided intracranial ICA, MCA, and ACA with slight reflux to the left side and posterior circulation (Figure 1).

Supplementary Material Video S1: Real-time dynamic EPI-GRE MRI during test infusion of diluted gadolinium through microcatheter.

## 3. Results

The anesthetized pig/swine with catheters secured was transported to 3T MRI (GE Ingenia).

After the series of anatomical T1 and T2 sequences, a dynamic gradient-echo (GE)—echo-planar imaging (EPI) scan was used to monitor microcatheter injection in real-time. Test infusion of diluted gadolinium (10 mM) was seen as a short wash-in and wash-out of hypointense signal, confirming the proper position of the microcatheter and verifying the area of trans-catheter perfusion (Supplementary Material Video S1). After filing the dead space of Apollo with 0.3 mL of DMSO, 3 mL of Onyx 18 (Medtronic) was injected.

**Figure 2:**
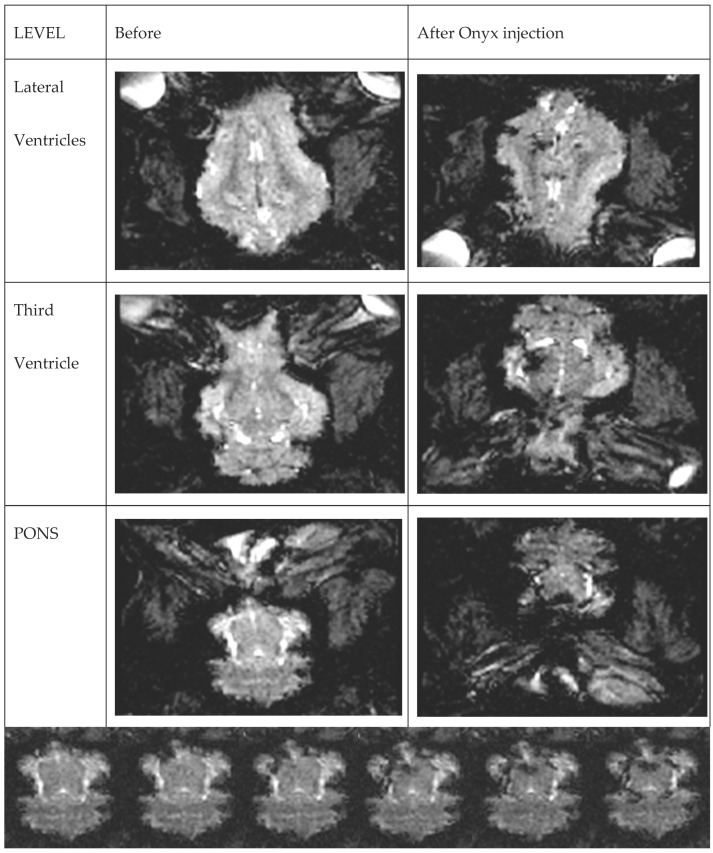

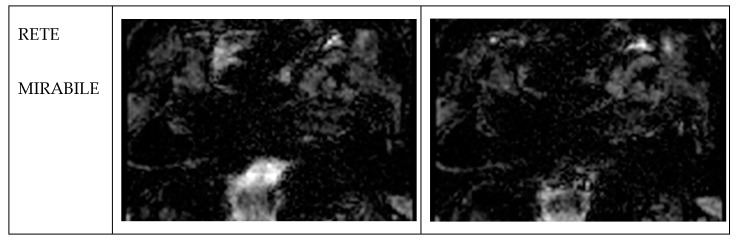
Real-time, dynamic EPI-GRE T2 scan of the whole brain volume during Onyx injection. Images on different levels before and after injection. The gradual filling is presented in Supplementary Material Video S2.

Onyx propagation was observed in real-time with initial ipsilateral RM filling followed by some main cerebral arteries and their branches distribution. The relatively bright signal within RM and the brain prior to Onyx injection provided a good background for the dark, low signal of the embolic agent spreading in rete mirabile and small brain arteries. (Supplementary Material Video S2/Figure 2).

Supplementary Material Video S2: Real-time, dynamic EPI-GRE T2 scan of the whole brain volume during Onyx injection. Cine sequence of the Onyx propagation at the level of the pons. Dark liquid embolic agent is gradually filling small arteries around the pons.

**Figure 3:**
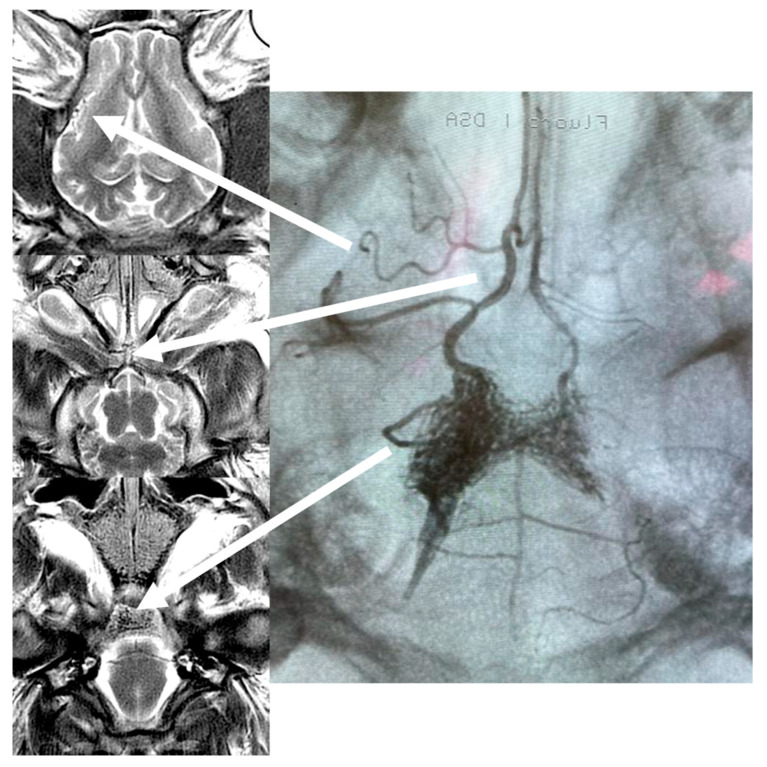
FU T2 scans 20 min after Onyx injection and an X-ray image of the Onyx cast.

T2 anatomical sequences and X-ray picture confirmed Onyx cast distribution at the end of the procedure (Figure 3).

## 4. Discussion

The long-term success of curative endovascular AVM embolization ultimately depends on the complete occlusion of the nidus, feeding arteries, and draining veins without disrupting any functional vessels of the brain [4]. In high grades IV and V (Spetzler–Martin Grade) AVMs, it is very difficult, if not impossible, to achieve these goals in one therapeutic session. As a consequence, more often than not, only part of the nidus is occluded in one treatment stage [4]. The foot of the draining vein, which should be occluded in the last order with transarterial embolization, is usually located in the central part of the nidus, and its visibility decreases while the nidus and feeding arteries are opacified with an embolic agent. This problem is even more pronounced in cases of multicompartmental bAVMs, where several feeders and draining veins can be divided into compartments connected or separated by small, nonfunctional, or even functional brain parenchyma [11]. Premature occlusion of the vein with the embolic agent before filling of the nidus can lead to thrombosis of this compartment, pseudo-occlusion, or contrast-stagnation on the DSA, and, last but not least, delayed bleeding after the procedure [7,8,9,10]. The angiographic resolution of AVM during liquid embolic injection, in our opinion, is not sufficient to claim embocure. We almost always progress with nidus embolization if possible, despite no obvious flow of contrast through AVM remaining in angiography. Injection of liquid embolic should be stopped in case of catheter occlusion or unnecessary reflux to feeding arteries or through arterio-arterial anastomoses. That said, an embocure can be declared once sufficient occlusion of feeding arteries, nidus, and draining veins occurs, often quite far beyond angiographic occlusion. Relying only on the angiographic cessation of the flow through AVM may be misleading with early recurrence or post-procedural bleeding, which we previously experienced in over 15 years of our practice with endovascular embolization of AVM. Moreover, there has been a significant number of patients around the world with partially embolised AVMs of all sizes in the past, waiting for the final treatment. The new method—MRI-guided embolization could allow for the precise targeting of “hidden compartments” and their blood supply, thus preventing AVM recurrence [9]. As a consequence, efforts are increasingly being made to develop tools and technologies to monitor endovascular neurointerventions with MRI. One such example is a report on multiplanar vascular navigation using a magnetically assisted remotely controlled catheter with real-time MRI [30]. Real-time MRI monitoring of intra-arterial infusions in the brain has been proven to be feasible both in animal models [31,32] as well as in the first-in-human application [33,34]. Contrast media used in cited publications (feraheme or diluted gadolinium) caused a transient signal drop in vessels which is detected in dynamic perfusion sequences [31,32,33,34]. Similar properties of liquid embolic agent—Onyx on MRI signal were used in our experiment. One of its main ingredients, i.e., tantalum powder, opacifies X-rays and causes the signal drop in MRI. Opposite to MRI contrast media embolic agents are solidifying and cause durable occlusion of the vessels, which is very well visualized in real-time during the procedure and on 20 min post-injection T2 scans (Figure 3). 

The contrast mechanism of Onyx in MRI sequences is based on its chemical composition and physical properties. Onyx is a mixture of dimethyl sulfoxide (DMSO), ethylene vinyl alcohol (EVOH), and tantalum powder. DMSO has a low signal intensity on both T1- and T2-weighted images, while EVOH has a high signal intensity on T2-weighted images. In T1-weighted images, Onyx appears as a low signal intensity due to the short relaxation time of DMSO. T2-weighted images, on the other hand, show a high signal intensity due to the long relaxation time of EVOH. The addition of tantalum powder to Onyx provides a source of susceptibility contrast, which enhances the visibility of the embolic agent in the MRI.

For our procedure, the most important imaging property of Onyx is significant susceptibility artifacts caused by the presence of tantalum powder. They can be detected on gradient-echo sequences such as susceptibility-weighted imaging (SWI) or T2*-weighted imaging (T2*WI), which are used for real-time MRI perfusion imaging.

Overall, the contrast mechanism of Onyx in MRI sequences is based on the combination of its chemical composition and physical properties, which result in distinct signal intensity patterns on T1- and T2-weighted images and susceptibility artifacts on gradient-echo sequences.

Although the detection of Onyx in meningeal arteries might be problematic due to the proximity of low signal tissues like bone, GE-EPI sequences show high sensitivity for the detection of Onyx propagation in the vessels surrounded by the brain and CSF. As much as the temporal resolution of perfusion sequences used in this experiment is inferior to X-ray imaging, the significant advancements in dynamic MRI allow for acquisition times of serial images of 20–30 ms, which correspond to MRI videos with rates of up to 50 acquisitions per second, offering perfusion sequences far superior to X-ray fluoroscopy [16,17,18]

The rationale for employing real-time MRI is to reduce the rate of complications and improve the rate of total occlusions [9,10]. Intraprocedural rupture with bleeding during liquid embolic infusion was not explored in our experiment, and it obviously should be included in the next experiments. Theoretically, the real-time gradient echo sequence used for Onyx infusion monitoring is probably the most accurate to visualize acute bleeding, and it should be visible as a low signal intraparenchymal hematoma. To our best knowledge, a similar problem may occur during MRI monitoring of focused ultrasound (FUS) thalamic destruction or laser interstitial therapy [35,36,37].

We believe that using more precise volumetric acquisition in the future may significantly improve the degree of AVM occlusion and thus increase the efficacy of endovascular treatment. Moreover, this kind of data is much easier to employ in robotic surgery, machine learning, and AI algorithms. That said, we have been strongly driven to explore the potential possibilities of MRI monitoring of Onyx infusions. We have considerable experience with this technique. We have previously used RT-MRI to monitor iron-labeled stem cells during intra-arterial infusions in swine and dog models [27], as well as inducing stroke in swine [28]. These findings have been translated into clinical practice as we have shown the feasibility of using RT-MRI for intra-arterial administration of bevacizumab in glioma patients [29,31]. Although the technique is far from replacing the traditional method of using X-rays in monitoring liquid embolic agents, it has demonstrated a significant potential with better delineation of Onyx spatial distribution in real-time.

Given that we have only relied on Onyx, other liquid embolic materials available, such as SQUID (Balt, Montmorency, France), Precipitating Hydrophobic Injectable Liquid (PHIL©; Microvention, Tustin, CA, USA), or Eudragit-E 100 (Röhm America, Piscataway, NJ, USA), also require studying in the MRI setting.

### 4.1. Presentation of the Image Data

An X-ray image during AVM embolization is displayed as a sum of shadows of various tissues, catheters, and embolic agents with different opacities. Spatial 3D or even 4D data are acquired during a rotational scan at the beginning of the procedure and can be used as a landmark or live roadmap. Still, they are not true real-time images, and in case of any distortion of anatomy, the acquisition must be repeated. The whole distance from the X-ray lamp to the detector is visualized, but some of the projections (e.g., along the long axis of the patient) cannot be obtained; however, even with the latest biplane technology we cannot see through a large embolic agent (e.g., Onyx) cast due to the high level of opacification of the tantalum powder. On the other hand, MRI is a volumetric scanning technique involving signals from precisely limited space—an area of tissue with distinct borders like a cubic square or cylinder. Presentation of the data can be on any selected plane or as a volume rendering with different settings. As we previously published (in the first-in-human application of real-time MRI-guided endovascular neurointervention), semi-transparent reconstruction, which can be freely rotated by the operator, seems to be the most useful type of data presentation [29,31]. This type of display is also used in real-time MRI monitoring of the heart ablation procedures with active tracking of the catheter tip position and automatic adjustment of MPR and 3D [19,20].

### 4.2. Animal Models

The last decades have witnessed a concerted effort of the scientific community to develop in vivo and in vitro arteriovenous malformation models. This has resulted in numerous bAVM models, each with different advantages and disadvantages, as well as purposes [21,22,23,24,25,26,27,28,29].

For its network of microarteries that are interconnected and angiographically resemble an AVM nidus, rete mirabile was recognized early on as a potentially suitable AVM model, with many researchers eagerly employing it for endovascular studies [21,22,23,24,25,26,27,28,29]. However, it is characterized by low-flow, high-resistance arterial–arterial connections, lacking the arteriovenous shunting present in human AVMs. For this reason, many studies evaluating endovascular techniques have modified the model to adequately reflect hemodynamic conditions in humans. Chaloupka et al. created a short-term arteriovenous shunt between the rete, the internal carotid artery, and the cavernous sinus [23]. Unfortunately, such a model leads to several adverse effects on the animals, such as proptosis, chemosis, and sub-conjunctival hemorrhage, and lasts for a maximum duration of only a week as the connections occlude spontaneously [21,22,23,24]. In order to simplify the creation of the model and resolve the limitation of adverse effects of the rete-cavernous fistula model, Massoud et al. created an AVM model in the neck of the swine where three arteries are occluded in the neck region to increase, followed by a side-to-side anastomosis forming a carotid-jugular fistula [16]. Although this model has been successfully used in training and education, it is limited by the time-consuming and technically demanding creation of an arteriovenous fistula as well as the need for endovascular occlusion of at least three vessels. Siekmann et al. proposed a similar albeit less time-consuming and technically demanding model that does not involve the occlusion of vessels [25]. Given that we have evaluated the ability of real-time MRI to visualize the liquid embolic agent (Onyx) injection in a swine AVM model without assessing any hemodynamic properties, we did not introduce any modifications. In future animal experiments, anesthesia with complete muscle paralysis is necessary because injection of Onyx to rete causes significant contraction of neck/pharyngeal muscles resulting in motion artifacts.

Swine models are commonly used because of their large neck vessels and their similar coagulation system to humans, whereas rodent and dog models involving an arteriovenous shunt have been used to study the biology of arterialized veins, to investigate the effects of cerebral hypoperfusion or, to study the molecular and morphological changes in AVMs treated with radiosurgery [22,23,24,25].

Recently, Kaneko et al. have introduced a 3D printed model based on 3D rotational angiography from a patient. This model resolves the inherent limitations of in vitro AVM models, such as the lack of complex angioarchitecture characteristics of human bAVMs [21]. As much as there is no ideal AVM in vitro model, the proposed 3D printed model with feeders and draining veins attached to peristaltic pumps might prove to be adequate for the evaluation of real-time Onyx injections, avoiding the need for researchers to rely on in vivo models [21]. According to the authors, the injection of liquid embolic agents performed in the 3D printed model allowed for the replication of the plug and push technique before penetration of the liquid embolic material into the AVM nidus [21].

## 5. Conclusions

This is a preliminary report on the use of real-time MRI for visualizing embolic agent—Onyx propagation in the swine rete mirabile and cerebral arteries that resemble human brain AVM angioarchitecture. Onyx itself provides MRI imaging contrast well, mainly due to susceptibility artifacts caused by its ingredient—tantalum powder, which sets embolized vessels apart from brain tissue and cerebrospinal fluid. Although we have shown that it is possible to successfully use this technique for the first time, significant work both in vitro and in vivo regarding dedicated sequences and data display is paramount for the further development of this technique.

## Data Availability

The datasets generated and/or analyzed during the current study are available from the corresponding author upon reasonable request.

## Supplementary Materials

The following supporting information can be downloaded at: https://www.mdpi.com/article/10.3390/brainsci13060915/s1, Video S1: Real-time dynamic EPI-GRE MRI test infusion of diluted microcatheter under X-ray gadolinium. Video S2: Real-time, dynamic EPI-GRE T2 scan of the whole brain volume during Onyx injection.
